# Acute Hypersensitivity Reaction With Maculopapular Exanthem and Angio-Oedema Following Low Dose Amitriptyline for Migraine Prophylaxis: A Case Report

**DOI:** 10.7759/cureus.91469

**Published:** 2025-09-02

**Authors:** Bhanu Priya Singh, Anna Javed, Sartaj Hussain, Ritesh Kumar

**Affiliations:** 1 Pharmacology, Shri Mata Vaishno Devi Institute of Medical Excellence, Katra, IND; 2 Pharmacology, All India Institute of Medical Sciences Vijaypur, Jammu, IND

**Keywords:** adverse drug reaction, amitriptyline, drug hypersensitivity, maculopapular exanthem, tricyclic antidepressants

## Abstract

We report a case of a 34‑year‑old woman who developed a generalized maculopapular rash accompanied by urticaria‑like pruritus, angio‑oedema, and mild dyspnoea. The reaction occurred approximately one hour after ingestion of her routine 10 mg dose of amitriptyline, following four weeks of therapy for migraine prophylaxis. Laboratory studies showed elevated erythrocyte sedimentation rate (ESR) and CRP with normal eosinophil counts and negative viral serology; imaging was unremarkable. Causality, assessed with the World Health Organization-Uppsala Monitoring Centre system (WHO‑UMC) and Naranjo algorithms, was “probable.” Prompt cessation of the medication, H_1_ receptor antagonism combined with leukotriene‑receptor blockade, and supportive treatment resulted in complete recovery by day eight, with no recurrence observed at the three-month follow-up. This case expands the limited literature on tricyclic antidepressant (TCA)-induced hypersensitivity and emphasizes the importance of timely recognition, discontinuation, and pharmacovigilance reporting for patient safety.

## Introduction

Amitriptyline, a prototypical tricyclic antidepressant (TCA), remains widely prescribed for migraine prophylaxis and chronic pain syndromes, in addition to its traditional role in depression management [1]. Its therapeutic effects are attributed to inhibition of serotonin and noradrenaline reuptake, alongside potent anticholinergic and antihistaminic properties [2]. While the anticholinergic and cardiotoxic effects of TCAs are well established and closely monitored, their cutaneous hypersensitivity reactions are uncommon, with an estimated frequency of ≤0.1% of exposed patients [3].

The clinical spectrum of TCA-induced hypersensitivity is broad, ranging from mild exanthematous eruptions and urticaria to severe presentations such as drug reaction with eosinophilia and systemic symptoms (DRESS) or Stevens-Johnson syndrome [4]. However, the published literature documenting such reactions to amitriptyline is sparse, with only a few isolated reports since the first detailed description in 2000 [5]. Given the increasing off-label use of amitriptyline for non-psychiatric indications, recognition of rare but potentially life-threatening hypersensitivity reactions is essential.

This case report was presented as a poster entitled 'Amitriptyline-Induced Acute Idiosyncratic Anaphylactic Syndrome: A Case Report' at the 3rd National Conference of the Association of Clinical Pharmacologists (ACPCON 2024), held in Mumbai, India, in September 2024.

## Case presentation

A 34-year-old woman with no history of drug allergy and not taking any other medicine for any illness had been on amitriptyline 10 mg for migraine prevention for one month. On day 0, roughly one hour after administration of the drug, she manifested generalized erythematous pruritic eruptions, angio‑oedema of the lips and tongue, dysphagia, dysarthria, and dyspnoea. She presented to the emergency department with tachycardia (heart rate: 130 beats/min), tachypnoea (respiratory rate: 25/min), and widespread erythroderma.

Physical examination

**Figure 1:**
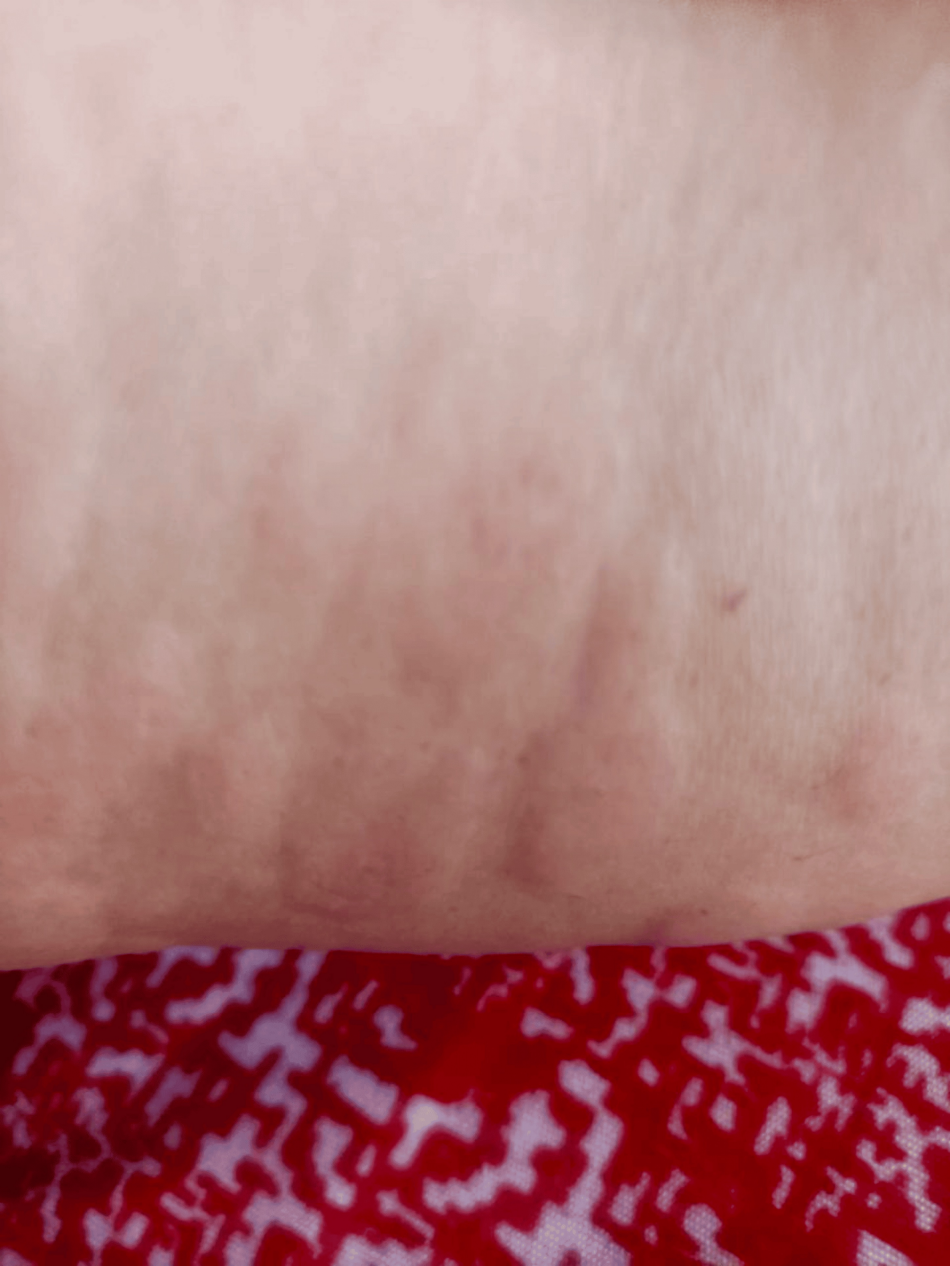
Diffuse pink macular erythema with oedema observed one hour after amitriptyline ingestion, indicating the acute onset of hypersensitivity

**Figure 2:**
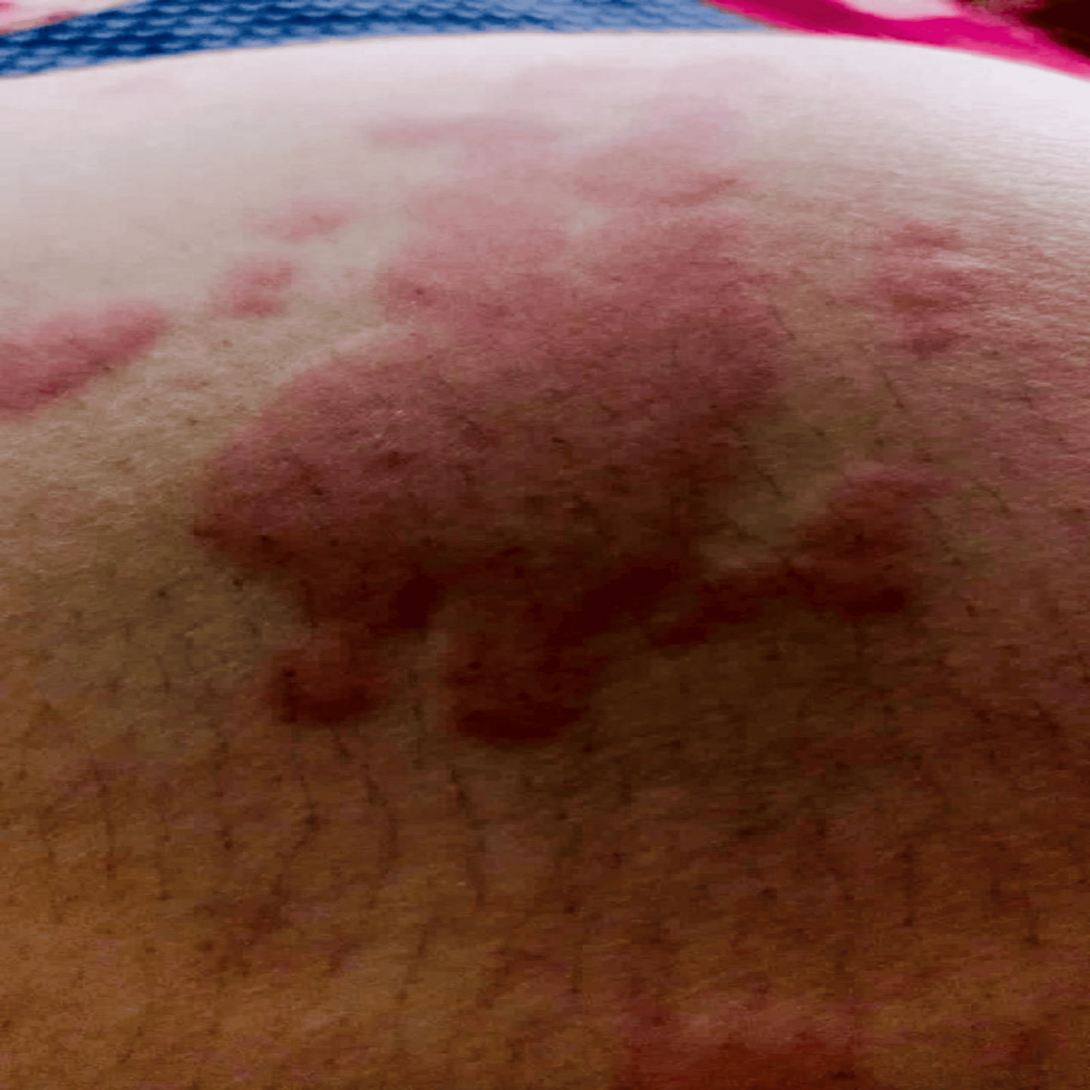
Coalescing red maculopapular lesions at 24 hours, showing progression of the eruption without mucosal involvement

**Figure 3:**
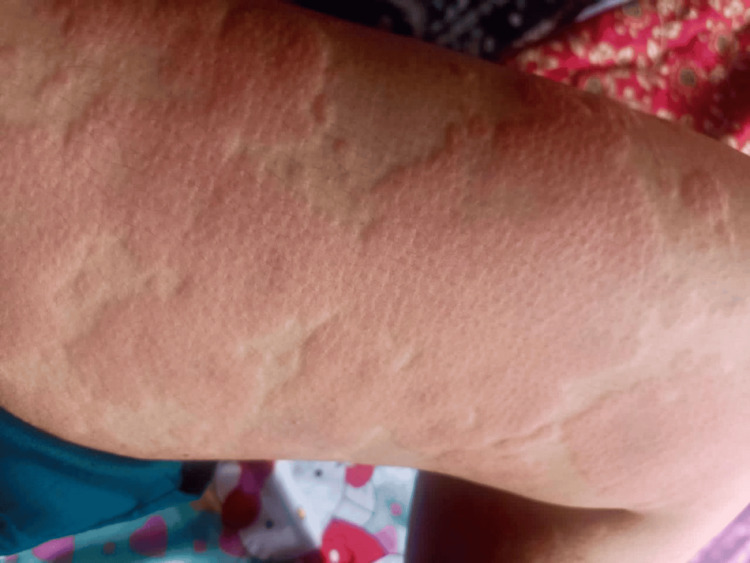
Hyperpigmented papules with fine scaling at 48 hours, consistent with resolving exanthem

Generalised macular erythema with oedema was evident at one hour after drug exposure, evolving to red maculopapular lesions by 24 hours, and to hyperpigmented papules with fine scaling by 48 hours (Figures 1-3); mucous membranes remained intact, with no mucosal erosions throughout the course.

The patient underwent various radiological and biochemical investigations to determine the cause (Table 1).

**Table 1:** Laboratory and nonrestrictive-laboratory investigations of the patient Urinalysis abnormalities were incidental and not related to the drug reaction. ESR: Erythrocyte sedimentation rate; HIV: Human immunodeficiency virus); HTLV-I/II: Human T-lymphotropic virus type I/II; CMV: Cytomegalovirus; EBV: Epstein-Barr virus.

Category	Test/Parameter	Result	Reference interval	Interpretation
Inflammation	ESR (mm/h)	30	0-20	Elevated
	CRP (mg/L)	9		Elevated
Hematology	Total leucocyte count (TLC; x10^9^/L)	Within reference limits	4.0-10.0	Normal
	Differential leucocyte count (DLC; %)	N_70_E_1_L_26_M_3_B_0_	N: 40-80; L: 20-40; M: 2-10; E: 1-6; B: <1-2	Normal
	Absolute eosinophil count (AEC; x10^9^/L)	0.08	0.02-0.50	Normal
Urinalysis	Colour	Dark yellow	Clear–pale yellow	Abnormal
	Appearance	Cloudy	Clear	Abnormal
	pH	4	4.5-8.0	Low/acidic
	Specific gravity	1.031	1.005-1.025	Elevated
	Cast, crystal, bacteria	Negative		
	Culture & Sensitivity	Negative		
Serology	HIV, hepatitis (A, B, C), HTLV-I/II, CMV, syphilis, and EBV	Non‑reactive	Non‑reactive	Negative
Non‑laboratory	ECG	Sinus tachycardia	—	Abnormal rhythm (rate >100 bpm)
	Chest X‑ray	Normal	—	Normal
	Abdominal ultrasound	Normal	—	Normal
	Echocardiogram	Normal	—	Normal

Differential diagnosis

The differential diagnosis in this case included (1) Drug‑induced maculopapular exanthem with angio‑oedema; (2) Viral exanthem (ruled out by negative serology); (3) Acute autoimmune urticaria (less likely due to close temporal relation to drug); and (4) Drug Reaction with Eosinophilia and Systemic Symptoms or DRESS (no eosinophilia, organ involvement)

Treatment

Amitriptyline was discontinued immediately. Therapy comprised oral desloratadine 5 mg plus montelukast 10 mg twice daily and topical calamine-aloe lotion. Cefixime‑clavulanate was used as empirical treatment for the abnormal urine analysis, with precaution against secondary skin infection.

Outcome and follow‑up

By day eight, the eruption had stopped progressing, showed diffuse desquamation, and systemic symptoms had resolved. No relapse was reported at the three‑month telephonic follow‑up. Amitriptyline was the exclusive suspect; no alternative sickness, medicine, or chemical could account for the occurrence. The re-challenge was not conducted due to ethical considerations. The reaction was Hartwig level 3 (moderate) - required emergency intervention but caused no permanent harm - and classified as “potentially preventable” on the Schumock-Thornton scale [6]. Both the the World Health Organization-Uppsala Monitoring Centre (WHO‑UMC) and Naranjo algorithms rated causality as “probable” [6]. The incident was reported to the National Coordination Centre-Pharmacovigilance Programme of India (PvPI) within 24 hours. Based on the clinical features and temporal profile, this reaction is best classified as a Type B (idiosyncratic, immunologic/hypersensitivity) adverse drug reaction [7].

## Discussion

Cutaneous adverse drug reactions (CADRs) to psychiatric medications are acknowledged but rather infrequent [8]. A 13-year pharmacovigilance study, including 2,08,401 inpatients suffering from mental disorders, identified clinically significant CADRs in only 0.1% of all exposures, with traditional tricyclic and tetracyclic antidepressants presenting a greater risk than newer antidepressants [9]. However, vigilance is crucial because rapid-onset urticaria can lead to anaphylaxis, as happened to our patient who developed cutaneous wheals, angio-oedema, and airway compromise within an hour of ingestion.

Amitriptyline exhibits strong H₁ blockade yet can paradoxically elicit a hypersensitivity reaction. Proposed explanations include direct mast cell degranulation, reactive metabolite haptenation, and hereditary vulnerability to human leukocyte antigen (HLA) [10,11]. The temporal relationship, resolution upon withdrawal, absence of viral markers, and “probable” scores on both WHO‑UMC and Naranjo algorithms collectively support drug causality. DRESS was excluded by the absence of eosinophilia or systemic organ involvement. Immediate cessation and dual H₁/H₂ antagonism are the primary treatments for acute drug-induced urticaria; short-term systemic corticosteroids are reserved for progressive or resistant cases. Timely notification of national pharmacovigilance programmes is crucial for signal discovery, and our report contributes to the limited literature. Only a few cases of amitriptyline-induced urticaria or hypersensitivity have been documented since the initial comprehensive description in 2000 [5,12,13]. A limitation is that confirmatory allergy testing (serum tryptase, IgE, or patch testing) was not performed due to resource constraints, but the temporal relation, symptom resolution, and ‘probable’ causality scores still support drug attribution.

## Conclusions

This case highlights that, although cutaneous hypersensitivity to amitriptyline is rare, such reactions may arise abruptly, affect multiple systems, and pose serious risks. Early recognition and immediate discontinuation of the culprit drug remain the cornerstone of management, supported by symptomatic therapy. Our patient’s complete recovery without relapse demonstrates the importance of timely intervention. Adverse cutaneous reactions to psychotropic drugs are well documented, and existing principles emphasize that early identification and rapid intervention are critical to minimizing morbidity and mortality. While our case did not progress to severe phenotypes such as Stevens-Johnson syndrome or toxic epidermal necrolysis, the acute onset of angio-oedema and respiratory compromise highlights the potential for escalation. Finally, systematic pharmacovigilance reporting remains essential to expanding the evidence base for rare ADRs. This report contributes to the limited literature on amitriptyline-induced hypersensitivity and reinforces the need for careful risk-benefit assessment, clinician vigilance, and patient education when initiating TCAs for migraine prophylaxis.
